# Supplementary material for: Interleukin 6 (IL-6) Regulates GABAA Receptors in the Dorsomedial Hypothalamus Nucleus (DMH) through Activation of the JAK/STAT Pathway to Affect Heart Rate Variability in Stressed Rats
Source: Int J Mol Sci. 2023 Aug 19;24(16):12985. doi: 10.3390/ijms241612985 (PMC10455568; doi:10.3390/ijms241612985)
Supplement: Supplementary file 1 [file ijms-24-12985-s001.zip › ijms-2536859-SI.pdf]

SUPPLEMENT MATERIALS AND METHODS

Behavioral testing

Behavioral experiments in all groups of rats were performed on the day after the model was established.

Open-field test (OFT) Rats were gently placed in the center of an acrylic box (100 cm ×100 cm × 40 cm) and allowed to freely explore the area for 6 min. The track of each rat was recorded with a video camera and subsequently analyzed using a spontaneous activity video analysis system (Shanghai Jiliang Software Science&Technology Co., Ltd., Shanghai, China). The metrics were used as measures of anxiety-like behavior, including the total distance, the distance traveled in the center and the percentage of cumulative time in the center area [(Cumulative time in the center area/Cumulative time in the overall area) × 100].

Elevated plus-maze (EPM) The EPM consisted of two open arms (50 cm×10 cm), two closed arms (50 cm×10 cm×40 cm) and a central area (10 cm×10 cm). The Plexiglas arms were elevated 50 cm above the ground. Behavioral testing was performed under dim red lights (28 lux). The track of each rat was recorded by an overhead camera, and the behavior of rats was scored for 5 min by a spontaneous activity video analysis system (Shanghai Jiliang Software Science&Technology Co., Ltd., Shanghai, China). The percentage of time spent in the open arms {[Time in open arms/(Time in open arms+Time in closed arms)]×100} and the percentage of open arm entries{[Open arm entries/(Open arm entries+Closed arm entries)]×100} were used as measures of anxiety-like behavior, and the number of closed arm entries was used as a measure of general locomotor activity.

SUPPLEMENT RESULTS

*Stress caused changes of body weight and anxious behavior in rats***.**

Changes in body weight and behavior are good physiological indicators of stress. Previous studies have shown that stress could cause weight loss and lead to anxiety-like behavioral changes in rats. As shown in Figure 1A, the body weight of stress rats gained slowly compared with the control group. OFT and EPM were internationally recognized behavioral methods for measuring the anxiety response of rodents. In the OFT, ANOVA revealed that stress treatment led to significant effects on the distance traveled in the center (Figure 1C) and the percentage of cumulative time spent in the center (Figure 1D), while there is no difference in total movement distance(Figure 1B). Post hoc comparisons showed that the distance traveled in the center and the percentage of cumulative time spent in the center of rats were significantly decreased after 3d (P<0.01; P<0.05), 7d (P<0.01; P<0.01) and 21d (P<0.01; P<0.05) of stress exposure. In the EPM, ANOVA showed that stress treatment led to significant effects on the percentage of entries into the open arms (Figure 1E) and the percentage of time spent in the open arms (Figure 1F). The post hoc test indicated that the percentage of entries into the open arms (P<0.05) and the percentage of time spent in the open arms (P<0.01) of stressed rats reduced significantly compared with the control group. The changes in body weight and behavior of rats suggested that the stress rat model has been successfully established.

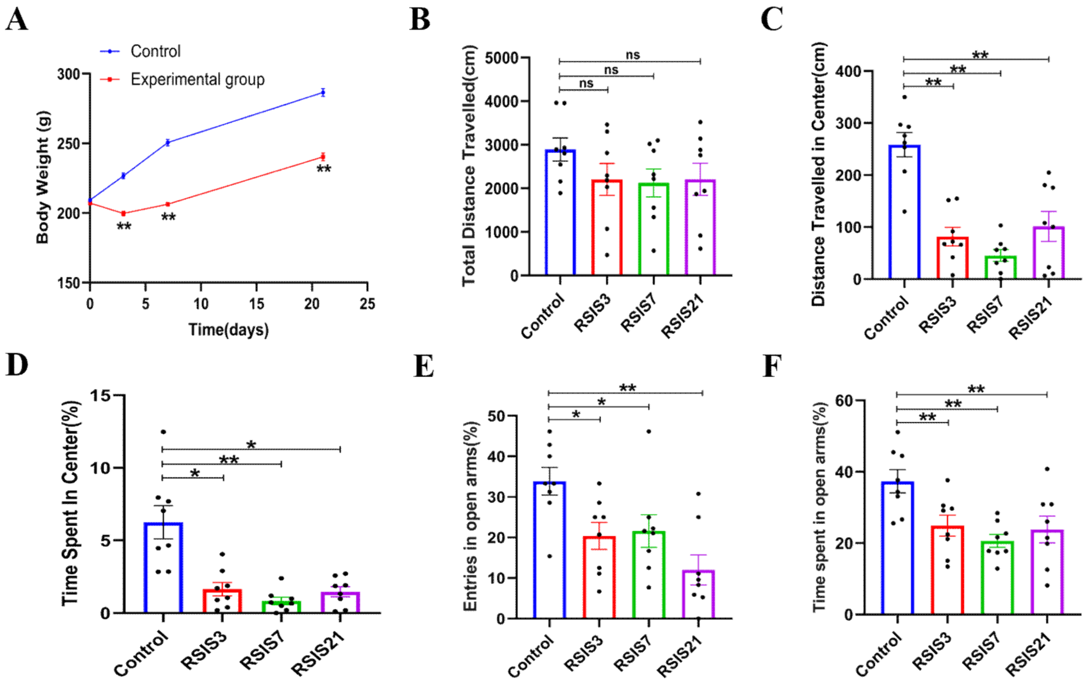

Supplementary Figure S1 Stress caused changes of body weight and anxious behaviors in rats. (A) Weight change. Stress significantly decreased the weight of rats compared with the control group. (B-D) Changes in anxiety-like behavior in the OFT after stress exposure. The distance traveled in the center and the percentage of cumulative time in the center were significantly decreased after stress exposure, while the total distance traveled did not change significantly. (E-F) Changes in anxiety-like behavior in the EPM after stress exposure. The percentage of entries in open arm and the percentage of time spent in open arms were markedly lower after stress exposure. Values are expressed as the mean ± SEM, $^{*}P<0.05$, $^{**}P<0.01$ vs. the control group, n=8.
